# THE CENTER FOR EQUITY IN AGING AT JOHNS HOPKINS UNIVERSITY

**DOI:** 10.1093/geroni/igae098.0727

**Published:** 2024-12-31

**Authors:** Katherine Ornstein, Sarah Allgood, Kali Thomas, Quincy Samus

**Affiliations:** Johns Hopkins University, Baltimore, Maryland, United States; Johns Hopkins University, Baltimore, Maryland, United States; Johns Hopkins University, Baltimore, Maryland, United States; Johns Hopkins University, Baltimore, Maryland, United States

## Abstract

The Center for Equity in Aging was launched in 2022 Johns Hopkins University to advance scholarship, research and advocacy focused on aging and serious illness care. The Center seeks to transform the diverse experiences of aging and serious illness to enhance equity and well-being across the life course. Hosted by the School of Nursing, the Center includes 2 dozen principal faculty and draws upon a variety of disciplines including nursing, epidemiology, gerontology, health services, and health policy. In this presentation we will discuss how the Center advances equity in aging by engaging in research, training and scholarship that supports diverse individuals and populations, identifies health disparities in aging throughout the life course, and develops interventions, policies, and models of care to reduce inequities in the opportunities and resources needed to grow and age as healthily as possible. We will discuss the Center’s collaborations and partnerships with a diverse set of stakeholder groups to promote community-engaged research practices that inform research across all stages and describe new initiatives focused on advancing equity within the Center and how the Center collaborates with other centers/institutes both within and outside of the Johns Hopkins community. Center leadership will discuss their visions for promoting equity in aging research and reflect on key challenges in the field.

